# EDP-938, a novel nucleoprotein inhibitor of respiratory syncytial virus, demonstrates potent antiviral activities *in vitro* and in a non-human primate model

**DOI:** 10.1371/journal.ppat.1009428

**Published:** 2021-03-15

**Authors:** Michael H. J. Rhodin, Nicole V. McAllister, Jonathan Castillo, Sarah L. Noton, Rachel Fearns, In Jong Kim, Jianming Yu, Thomas P. Blaisdell, Joseph Panarese, Brian C. Shook, Yat Sun Or, Bryan Goodwin, Kai Lin

**Affiliations:** 1 Enanta Pharmaceuticals Inc., Watertown, Massachusetts, United States of America; 2 Department of Microbiology, Boston University School of Medicine, Boston, Massachusetts, United States of America; ViiV Healthcare, UNITED STATES

## Abstract

EDP-938 is a novel non-fusion replication inhibitor of respiratory syncytial virus (RSV). It is highly active against all RSV-A and B laboratory strains and clinical isolates tested *in vitro* in various cell lines and assays, with half-maximal effective concentrations (EC_50_s) of 21, 23 and 64 nM against Long (A), M37 (A) and VR-955 (B) strains, respectively, in the primary human bronchial epithelial cells (HBECs). EDP-938 inhibits RSV at a post-entry replication step of the viral life cycle as confirmed by time-of-addition study, and the activity appears to be mediated by viral nucleoprotein (N). *In vitro* resistance studies suggest that EDP-938 presents a higher barrier to resistance compared to viral fusion or non-nucleoside L polymerase inhibitors with no cross-resistance observed. Combinations of EDP-938 with other classes of RSV inhibitors lead to synergistic antiviral activity *in vitro*. Finally, EDP-938 has also been shown to be efficacious *in vivo* in a non-human primate model of RSV infection.

## Author summary

Respiratory syncytial virus (RSV) is a ubiquitous viral pathogen which inflicts a significant healthcare burden and is responsible for thousands of deaths annually. Currently no vaccine or targeted therapeutic exists. This work characterizes a newly discovered small molecule inhibitor of the virus, EDP-938, whose activity is mediated through the viral nucleoprotein. EDP-938 has potent *in vitro* activities against laboratory strains and clinical isolates of the virus, presents a high-barrier to resistance, can work synergistically with other known fusion or L protein inhibitors, and displays strong *in vivo* efficacy in a non-human primate model for RSV infection. EDP-938 is currently under evaluation in Phase 2 clinical studies.

## Introduction

Respiratory syncytial virus (RSV) is a ubiquitous viral pathogen which can repeatedly re-infect a person throughout their lifetime. While most healthy children and young adults suffer mild symptoms with upper respiratory tract infections, progression to more serious lower respiratory tract infections do occur and RSV with significant mortality reported in at-risk groups such as infants, the immunocompromised, and the elderly [1–3]. RSV is the leading cause of respiratory induced hospitalizations, especially in children under 5, and is responsible for an estimated 3.4 million hospitalizations globally and 95,000–150,000 deaths per year [4,5]. In lower-middle income countries the majority of RSV-related fatalities occur in community settings, suggesting an underestimation of RSV’s true global impact [1,6].

Current prophylactic and therapeutic options for RSV are limited. Despite ongoing development efforts there is no approved vaccine or direct-acting antiviral against RSV. Treatment options include supportive care and the broad-spectrum antiviral ribavirin, whose usage is limited due to questionable efficacy and side effects [7–9]. For premature infants who are 6 months of age or younger at the start of the RSV season, the monthly injectable monoclonal antibody palivizumab is available, providing approximately 55% relative reduction in RSV-associated hospitalizations [10].

RSV is a non-segmented negative strand RNA virus of the family *Pneumoviridae*. Divided into subgroups A and B, its 15kb genome consists of 10 genes encoding 11 proteins. Transcription and replication involve the ribonucleoprotein complex which consists of the viral RNA genome or antigenome encapsidated by nucleocapsid N proteins. This structure provides protection from cellular degradation and acts as a guide for the RNA-dependant RNA polymerase L protein with assistance from P and M2-1 proteins [11].

A number of small molecule inhibitors directly targeting RSV have been identified. The majority of these compounds, known as fusion inhibitors, bind to the surface F protein, thus preventing viral entry into the cell. The first fusion inhibitor to show antiviral activity in a human RSV challenge study in healthy volunteers was presatovir (GS-5806) [12]. However, in subsequent phase 2 clinical trials presatovir failed to meet its virologic and clinical endpoints across a range of patient cohorts [13–15]. Additionally, resistance-associated mutations were selected quickly in patients receiving presatovir, suggesting a low barrier to resistance with this molecule [16,17]. Several other fusion inhibitors, JNJ-53718678 [18], ziresovir (AK0529) [19] and sisunatovir (RV521) [20], are currently under clinical development for RSV. Compounds targeting post-entry viral replication have also been described including the nucleoside inhibitor lumicitabine (ALS-8176) [21], non-nucleoside L polymerase inhibitors AZ-27 [22], PC786 [23] and JNJ-64417184 [24]. Among them, lumicitabine showed proof-of-concept efficacy in a human RSV challenge study [25] but its development was later terminated [26,27].

Another potential interesting target for RSV is the viral nucleoprotein (N), which is the most conserved viral protein and is essential for both viral replication and transcription [11]. A series of 1,4-benzodiazepine analogues were identified as specific inhibitors of RSV through cell-based antiviral screen of a diverse library of 20,000 compounds [28]. Later it was proposed that viral N protein is the target of the inhibitors mainly through *in vitro* resistance study [29]. The lead molecule, RSV-604, was advanced into human studies and showed some promising antiviral activities in a subset of stem cell transplant patients whose drug level was above its *in vitro* 90% maximal effective concentration (EC_90_). However, the compound was later discontinued because of its suboptimal potency and the development challenge to achieve sufficient drug exposure [30].

Despite the challenges of development, there is a clear need for antiviral treatment options for RSV. EDP-938 was identified through a series of chemical optimizations based on 1,4-benzodiazepine inhibitors of RSV [31]. Here we report the *in vitro* and *in vivo* antiviral activities of EDP-938. EDP-938 effectively blocks RSV replication by targeting a post-entry replication stage of the viral life cycle. *In vitro* resistance studies confirmed that it targets the viral N protein. EDP-938 is currently under evaluation in Phase 2 clinical studies.

## Results

### *In vitro* activity of EDP-938 against RSV

**Fig 1:**
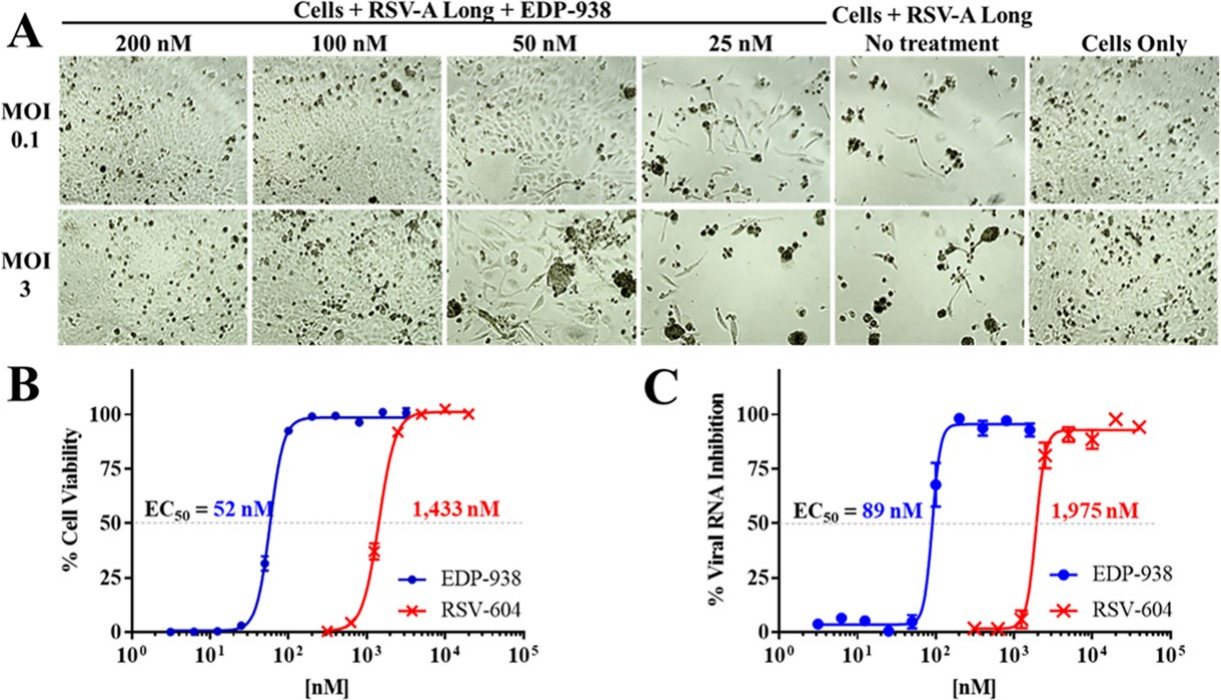
Inhibition of RSV *in vitro* by EDP-938. (A) Visualization of HEp-2 cell monolayers following RSV-A Long infection with or without treatment at indicated levels of EDP-938 with 5-day post infection (dpi) endpoint. (B) Percent cell viability measured by ATPlite and (C) percent viral RNA reduction measured by RT-qPCR in cells infected with RSV-A Long at MOI of 0.1. Data are mean ± standard error of the mean (SEM) from 89 and 77 individual experiments for EDP-938 and RSV-604, respectively, in the CPE assay and 8 separate assay runs for the RT-qPCR endpoint.

Based on reactivity of surface antigens to monoclonal antibodies and genetic variability, two subgroups of human RSV, A and B, have been delineated. Initially, the antiviral activity of EDP-938 was assessed in human epithelial cells (HEp-2) infected with RSV-A Long. As expected, visual inspection of the RSV-infected HEp-2 cell monolayers revealed an extensive cytopathic effect (CPE) in untreated cells. However, cells treated with EDP-938 exhibited a dose-dependent reduction in RSV-induced CPE. The cytoprotective effects of EDP-938 were independent of the RSV-A Long multiplicity of infection (MOI) (Fig 1A). To obtain a more quantitative measure of the antiviral activity of EDP-938, cellular ATP levels were determined. These data demonstrated that EDP-938 inhibited RSV induced CPE with a half-maximal effective concentration (EC_50_) of 52 ± 12 nM (Fig 1B and Table 1). Using a reverse transcription quantitative polymerase chain reaction (RT-qPCR) assay which monitors the reduction of viral RNA in the presence of compound, EDP-938 reduced viral load with an EC_50_ of 89 ± 16 nM. In line with previous publications [29], RSV-604 blocked RSV-A Long-induced CPE and inhibited viral replication with an EC_50_ of ~1.4 μM and ~2 μM, respectively (Fig 1C).

**Table 1:** *In vitro* activity of EDP-938 against RSV laboratory strains.

Cell	Virus Strain	Assay	EDP-938 EC_50_ [nM]	EDP-938 EC_90_ [nM]
			Mean ± SD	Mean ± SD
HEp-2	RSV-A Long	CPE	52 ± 12	60 ± 18
		RT-qPCR	89 ± 16	91 ± 62
	RSV-A2	CPE	28 ± 4	40 ± 3
		RT-qPCR	59 ± 18	70 ± 30
	RSV-A M37	CPE	28 ± 4	34 ± 10
		RT-qPCR	54 ± 5	85 ± 21
	RSV-B VR-955	RT-qPCR	110 ± 22	140 ± 24
A549	RSV-B VR-955	CPE	72 ± 9	223 ± 90
		RT-qPCR	83 ± 38	170 ± 53
Vero	RSV-A Long	RT-qPCR	79 ± 12	118 ± 25
	RSV-A2	CPE	33 ± 7	44 ± 18
		RT-qPCR	91 ± 0	101 ± 1.7
HBEC*	RSV-A Long	RT-qPCR	21 ± 17	29 ± 23
	RSV-A M37	RT-qPCR	23 ± 14	36 ± 17
	RSV-B VR-955	RT-qPCR	64 ± 30	74 ± 33
BHK	RSV-A Long	RT-qPCR	16,800	-
Cotton rat lung*	RSV-A Long	RT-qPCR	10,280	-
Neonatal lamb bronchial epithelial*	RSV-A Long	RT-qPCR	>800	>800

* Indicates primary cell line

The activity of EDP-938 against both RSV-A and RSV-B was evaluated *in vitro* against multiple laboratory strains commonly used for antiviral screens in HEp-2 cells, human adenocarcinomic alveolar basal epithelial cells (A549), monkey kidney-derived cells (Vero), and Baby Hamster Kidney cells (BHK). Serial dilutions of the compound were added to cells infected with RSV-A at a MOI of 0.1 or RSV-B at an MOI of 0.5 and treated for 5 or 6 days, respectively. The EC_50_ values ranged from 28–72 nM for inhibition of CPE and from 54–110 nM for reduction in viral load as determined by RT-qPCR (Table 1) for the primate cell lines. However, a significant reduction of potency was observed in the BHK cell line, a finding previously observed for RSV-604 as well [32]. Together, these data show EDP-938 potently inhibits both RSV-A and B in various human and non-human primate cell lines using orthogonal assay read-outs.

RSV primarily replicates in the ciliated epithelial cells on the superficial layer of the airways. Primary human bronchial epithelial cells (HBECs) can be cultured and infected with RSV *in vitro*, which provides a more physiologically relevant system to evaluate potential antiviral drug candidates. The activity of EDP-938 was determined in HBECs infected with several RSV strains at a MOI of 0.1. RSV-A Long and M37 infected cells were treated for 6 days, whereas RSV-B VR-955 infected cells were treated for 7 days. As shown in Table 1, EDP-938 potently inhibited all 3 RSV strains tested in HBECs. EDP-938 was also interrogated using primary airway cells derived from cotton rats and neonatal lambs. Mirroring the results of the BHK cells, EDP-938 depicted reduced efficacy in these non-primate cell lines. EDP-938 was assayed for cytotoxicity in HEp-2, A549, Chinese hamster ovary (CHO), human embryonic kidney (HEK)-293 cells and the primary HBECs. There was no significant cytotoxicity with EDP-938 in HEp-2, A549, HEK-293 or HBEC at up to 50 μM, and the half-maximal cytotoxic concentration (CC_50_) in CHO cells was ~27 μM (S1 Table). This measurement yields a selectivity index (antiviral EC_50_/CC_50_) of >2,380 in HBEC cells. In addition, EDP-938’s antiviral activity is highly specific to RSV, as it did not demonstrate significant activity against a broad panel of other viruses (S2 Table).

**Fig 2:**
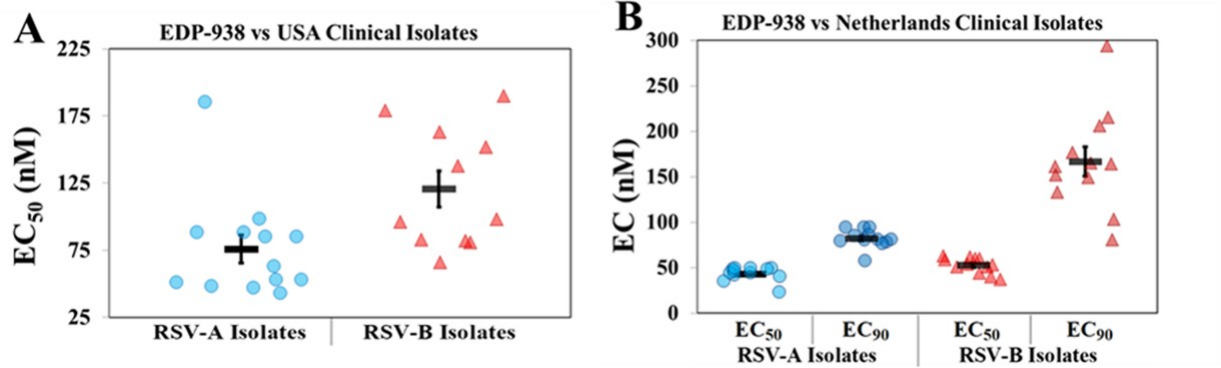
*In vitro* activity of EDP-938 against RSV clinical isolates. (A) Average EC_50_ of EDP-938 against RSV-A and RSV-B USA derived clinical isolates. (B) EC_50_ and EC_90_ (half maximal and 90% inhibitory concentration) of EDP-938 against 10 RSV-A and RSV-B clinical isolates from the Netherlands. Bold bars indicate the mean ± SEM.

To demonstrate that the anti-RSV activity of EDP-938 is not affected by the genetic variability of the virus, USA-derived clinical isolates were obtained from Baylor University and Medical College of Wisconsin and tested *in vitro* for susceptibility to EDP-938. Serial dilutions of the compound were added to cells infected with RSV-A isolates at an MOI of 0.1 (except for isolate 629-9-2 at an MOI of 0.01) or RSV-B isolates at an MOI of 0.5 (expect for isolate 629-24/2007 at an MOI of 0.0044) and were treated for 5 or 6 days, respectively. As shown in Fig 2A, EDP-938 was active against all the RSV-A and B clinical isolates tested with average EC_50_ values of 76 nM and 121 nM, respectively. This is consistent with the fact that the viral N protein, which is the putative target of the antiviral effect of EDP-938, is the most conserved RSV protein [11].

To further expand testing against clinical isolates, the compound was assayed against a panel of 10 RSV-A and 10 RSV-B strains mainly isolated from children of age 2–200 weeks in the Netherlands. EDP-938 had average EC_50_ values of 43 nM against RSV-A and 51 nM against RSV-B (Fig 2B) in the RSV Virospot reduction assay (ViroClinics).

The impact of protein-binding on the anti-RSV activity of EDP-938 was investigated *in vitro* in multiple cell types infected with various RSV strains. Unfortunately, human plasma or serum cannot be used directly in these cell-based studies because of the presence of RSV-neutralizing antibodies which interfere with the antiviral assay. Thus, purified human serum albumin or alpha-1-acid glycoprotein was added to the cell culture media at the physiologically relevant concentrations of 40 and 0.9 mg/mL, respectively. Albumin is the most abundant plasma protein whilst alpha-1-acid glycoprotein is known to be important in the binding and transport of many drugs. In this setting, a 2- to 5-fold shift in the potency of EDP-938 was observed in the presence of human serum albumin or alpha-1-acid glycoprotein across a range of RSV strains and cell types, suggesting that the effect of plasma protein binding may be minimal.

### *In vitro* resistance study

To understand the mechanism of action of the inhibitor and evaluate the potential impact of resistant variants, efforts were undertaken to generate and characterize drug resistant virus *in vitro*. RSV-A Long virus was passaged in the presence of increasing concentrations of EDP-938. The generation of EDP-938-resistant virus required a low starting concentration of drug relative to its *in vitro* EC_50_ (1x EC_50_). The concentration of EDP-938 was gradually increased (up to 64x EC_50_ in one resistance mutant) over 15 passages. Initiating viral passage at higher concentrations of EDP-938 or attempting to increase the concentration faster or higher resulted in clearance of the virus rather than selection of resistance. In contrast, the selection of resistance mutants using the fusion inhibitor GS-5806 [12] or L polymerase inhibitor AZ-27 [33] began at 10x their respective EC_50_ values and was quickly increased to 16,384x EC_50_ and 1,024x EC_50_, respectively (S1 Fig).

Five populations of RSV-A Long and two populations of RSV-B VR-955 drug-resistant virus were generated. The RSV-A viruses were plaque purified to derive multiple resistance clones (#1–7). The RSV-B-resistant viruses became non-cytopathic and, therefore, plaque purification could not be performed. As a result, viruses were tracked as populations using RT-qPCR. Plaque purified RSV-A viruses and RSV-B resistant populations were assayed for susceptibility to EDP-938 as well as RSV-604 and GS-5806 as controls. As shown in Table 2, two of the resistance clones conferred a 60-fold increase of EC_50_ against EDP-938. They also showed cross-resistance against RSV-604 but remained fully sensitive to GS-5806 as indicated by the lack of shift in compound potency. For each EDP-938 resistant strain, the complete genome was sequenced to identify mutations. All of the EDP-938 resistant viruses contain mutations in the RSV N protein (Table 2), which is consistent with previous resistance studies of structurally related compounds such as RSV-604 [29]. Interestingly, three of the resistant viruses also have mutations in the G glycoprotein, the function of which is unclear since G is responsible for viral attachment but not replication. The N protein is the most conserved protein of the virus while the G protein is the least [11]. It is possible that some of the G mutations selected represent compensatory mutations or random drift rather than conferring resistance. While GS-5806 and AZ-27 lost all ability to inhibit their resistant viruses (Table 2), these strains showed no signs of cross resistance when treated with EDP-938 or compounds targeting other mechanisms of action from those used to induce resistance.

**Table 2:** Characterization of EDP-938 resistant viruses.

Virus			Mutations in RSV N protein	Mutations in RSV G protein	EC_50_ Fold Change vs. WT		
					EDP-938	RSV-604	GS-5806
RSV-A Long	Wild-Type (WT)		-	-	1	1	1
	Plaque purified EDP-938 resistant clones	#1	Q102L, M109T, I129M	K205G, K213G, T219A	60	4.6	0.7
		#2	T29S, S134T		3.3	3.1	0.9
		#3	S134T		2.6	2	0.8
		#4	V90A, S134T		3.8	3.2	1.5
		#5	M109I	R8H	3.1	0.7	1.5
		#6	M109K		67	3.3	<0.23
		#7	K136R		2.7	2.2	1.2
RSV-B VR-955	Wild-Type		-	-	1	1	1
	Population 1		M109K, L139Q*		42	NA	0.2
	Population 2		M109T		6.6	NA	0.2

* Observed as a dual WT/Mutant population

To confirm the findings from the EDP-938 *in vitro* resistance studies, recombinant RSV viruses containing site-specific mutations in N were generated and tested against EDP-938, RSV-604, and GS-5806 in HEp2 cells. The key mutations identified in RSV N as shown in Table 2 were incorporated into recombinant viruses using site-directed mutagenesis in combination with an RSV reverse genetics-based approach. As shown in Table 3, mutations at N residues M109 and I129 caused the largest EDP-938 EC_50_ fold shifts relative to recombinant wild-type RSV. EDP-938 had an EC_50_ of ~1.2 μM against the M109K mutant, which corresponded to a 26.9-fold increase over its EC_50_ against the recombinant RSV wild-type. The EC_50_ against the I129M mutant was 170 nM, which corresponded to a 3.7-fold increase over wild-type. Recombinant viruses containing multiple mutations with N were also generated and tested. EDP-938 had an EC_50_ of ~2 μM against the recombinant virus harboring the Q102L/M109T/I129M triple mutants, which corresponded to a 42.4-fold increase relative to wild-type recombinant RSV and was comparable to the original resistance clone #1 selected with EDP-938 (Table 2). Moreover, recombinant RSV viruses containing the previously reported N mutations (I129L, L139I, and I129L/L139I) that confer resistance to RSV-604 [29] were also generated and evaluated. As shown in Table 3, the modifications at I129 and L139 produced RSV-604 shifts that were comparable to the previously published observations for RSV-604-resistant viruses [29]. Taken together, these findings support the observations from the *in vitro* EDP-938 resistance studies.

**Table 3:** Analysis of the impact of individual or combination of RSV N protein mutations on the antiviral activity of EDP-938 using the RSV reverse genetics system.

Mutations in RSV N protein	EC_50_ (nM)			EC_50_ Fold Change vs. WT		
	EDP-938	RSV-604	GS-5806	EDP-938	RSV-604	GS-5806
Wild-type (WT)	45 ± 21	1,153 ± 479	0.13 ± 0.12	1.0	1.0	1.0
Q102L	90 ± 10	1,338 ± 839	0.04	2.0	1.2	0.3
M109T	244 ± 61	1,862 ± 720	0.21	5.4	1.6	1.6
I129M	170 ± 18	5,063 ± 1,405	0.41 ± 0.40	3.7	4.4	3.2
Q102L/M109T	116 ± 68	2,426 ± 31	0.25 ± 0.18	2.6	2.1	1.9
Q102L/I129M	100 ± 6	3,228 ± 447	0.63 ± 0.65	2.2	2.8	4.8
Q102L/M109T/I129M	1,929 ± 817	6,409 ± 2,080	0.30 ± 0.26	42.4	5.6	2.3
V90A	46 ± 29	1,422 ± 430	0.13	1.0	1.2	1.0
M109I	70 ± 26	514 ± 159	0.11 ± 0.09	1.6	0.4	0.8
M109K	1,222 ± 610	2,867 ± 2,004	0.06 ± 0.05	26.9	2.5	0.5
S134T	99 ± 26	2,478 ± 245	0.23 ± 0.30	2.2	2.1	1.8
K136R	125 ± 20	4,299 ± 2,101	0.54 ± 0.34	2.7	3.7	4.2
N105D	60 ± 44	2,698 ± 221	0.14 ± 0.20	1.3	2.3	1.1
K107N	52 ± 6	1,812 ± 247	0.09 ± 0.07	1.2	1.6	0.7
I129L	233 ± 127	6,429 ± 3,098	0.12 ± 0.09	5.1	5.6	0.9
L139I	326 ± 83	9,552 ± 1,833	0.17 ± 0.15	7.2	8.3	1.3
I129L/L139I	626 ± 139	17,787 ± 4,317	0.10 ± 0.10	13.9	15.4	0.8

**Fig 3:**
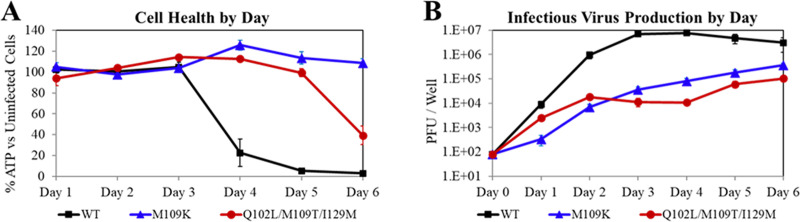
Growth kinetics of EDP-938 resistance mutants vs. wild-type (WT) virus. Viral Fitness analysis. (A) Cell viability compared to uninfected cells quantified by cellular ATP levels. (B) Plaque forming units (PFU) produced per well per day. Data are mean ± SEM, N = 3–7 for all runs.

It is important to note that all the EDP-938-resistant strains appeared to replicate at a much slower rate than the wild-type virus and showed less CPE in infected cells. The growth kinetics of two mutant viruses that conferred the highest levels of resistance against EDP-938 were assessed in a viral fitness assay compared to the wild-type virus. HEp-2 cells were infected with the mutant and wild-type virus at the same MOI of 0.01 and monitored over time. As shown in Fig 3 and S3 Table, the amount of replication-competent virus generated was ~100–400 times lower than wild-type, suggesting reduced viral fitness.

### Mechanism of action

**Fig 4:**
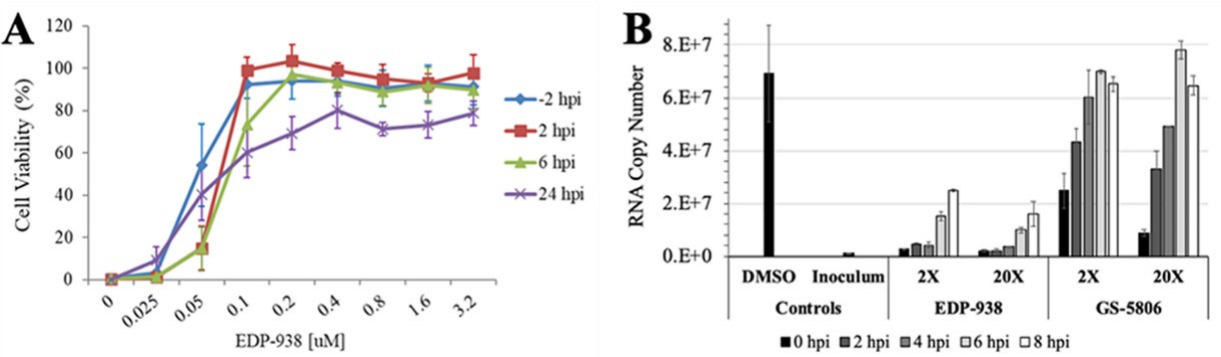
*In vitro* time-of-addition studies with EDP-938. (A) Cell viability of RSV-A Long-infected HEp-2 cells with EDP-938 treatment given at indicated times relative to infection. Cells were infected at an MOI of 0.1 and harvested 5 days post infection. All data are mean ± SD, N = 3–6 per time point. (B) Single-round RSV infection comparing EDP-938 and fusion inhibitor GS-5806 treated to untreated (DMSO) samples. A cold-synchronized infection was performed with virus at an MOI of 3. Viral RNA was quantitated by RT-qPCR at endpoint 24 hpi. 2x and 20x EC_50_ values are 100 nM and 1,000 nM for EDP-938, 2 nM and 20 nM for GS-5806. All data are mean ± SD, N = 2 per time point.

In order to help determine which step of the viral life cycle EDP-938 blocks multiple *in vitro* time-of-addition studies were performed. In the first study, EDP-938 was administered to cells infected with RSV-A Long at a MOI of 0.1 at 2 hours pre-infection (-2) or 2, 6, or 24 hours post-infection (hpi), and viral CPE evaluated 5 days later. As shown in Fig 4A, EDP-938 maintained about 80% of its cytoprotective activity for the cell population from RSV-induced CPE when given up to 24 hpi. The EC_50_ values of EDP-938 for cytoprotection were 49 nM, 55 nM, 76 nM, and 66 nM following compound addition at -2 h, 2 h, 6 h, and 24 h, respectively. A second time-of-addition study explored the ability of EDP-938 to protect not just a population experiencing a spreading infection, but individual cells already infected with RSV. Using a MOI of 3, which infects approximately 95% of all cells, virus was cold-adsorbed to the cells for 1 hour to synchronize infection before transfer to 37°C to induce fusion (marking the 0 hpi time point). Compound was added at times indicated and a 24 hpi RT-qPCR endpoint was employed to constrain viral replication cycle to a single complete cycle. As shown in Fig 4B, cells were either treated with DMSO, EDP-938 or the fusion inhibitor GS-5806 at either 2x or 20x their respective EC_50_. Compared to DMSO treatment, EDP-938 inhibited RNA production at all time points up to 8 hpi infection, however, compound efficacy began to diminishing at 6 hpi and beyond. In contrast, the antiviral efficacy of GS-5806 was reduced when added at 0 hpi and completely lost when added at 8 hpi (Fig 4B). These results suggest that EDP-938 works at a post-entry, replication step of the viral life cycle.

**Fig 5:**
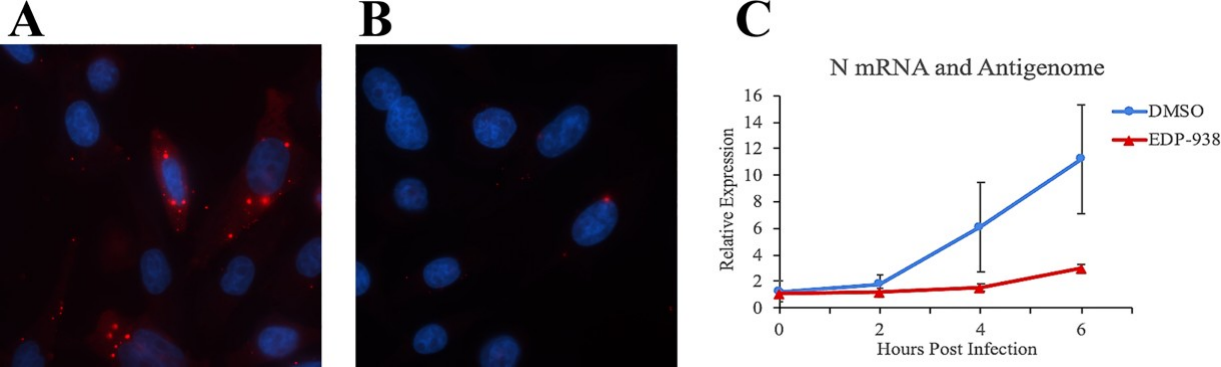
EDP-938 prevents primary transcription of RSV mRNAs. Following a synchronized infection with RSV-A2 (MOI of 3), DMSO or 1000 nM EDP-938 was added at 0 hpi. Immunofluorescence analysis of inclusion bodies at 8 hpi in the presence of DMSO (A) or EDP-938 (B). N protein was detected with an N specific monoclonal antibody (red) and cell nuclei were stained with DAPI (blue). (C) RT-qPCR analysis of RSV N gene expression between 0 and 6 hpi. Samples were quantified using the ΔΔCt method relative to the mean expression of housekeeping gene GAPDH. The relative expression of positive sensitive RNA is shown. Data are representative of two independent experiments.

EDP-938 inhibits an early stage of RSV replication following fusion. Following entry into a cell, nucleocapsids are transported from the plasma membrane into the cytoplasm, where viral transcription/replication “factories” are established. These “factories” manifest initially as small granules containing N, P, L, M2-1 and viral RNAs, and increase in size over time to form inclusion bodies [34,35]. Studies with RSV have shown that these inclusions can be formed with just the N and P proteins, and are dynamic liquid organelles which can readily dissociate and re-associate [36,37]. Given that resistance data suggest that N protein is the target of EDP-938, we investigated if the compound disrupted formation of the “factories”. RSV synchronously-infected HEp-2 cells were analyzed by immunofluorescence microscopy using an N-protein specific antibody. At 8 hpi, in the absence of compound, small RSV inclusions were detected in almost all cells (Fig 5A). In the presence of EDP-938, the majority of the cells did not present inclusions, however, in a few cells, inclusions were visible. These data suggest that EDP-938 did not inhibit liquid organelle formation per se (as they were present in some cells) but inhibited a prior step such that in most cells there was insufficient RSV protein for inclusion body formation.

To examine this more closely, we analyzed the effect of EDP-938 on accumulation of RSV RNAs. Again, HEp-2 cells synchronously-infected with RSV at 4°C were treated with either DMSO or EDP-938 upon transfer to 37°C (0 hpi). RNA isolated from DMSO treated cells showed that positive sense N RNA (likely representing N mRNA) increased significantly between 2 and 6 hpi (Fig 5C). However, in cells treated with EDP-938 transcription was inhibited with no significant increase above the background at any of the times post infection. These data indicate that EDP-938 inhibits RSV primary transcription and/ or processes prior to onset of primary transcription.

### *In vitro* combination study

The combination effect of EDP-938 with other RSV inhibitors of different mechanisms/targets was investigated *in vitro*. RSV-A Long infected HEp-2 cells were treated with various concentrations of EDP-938 either alone or in combination with the fusion inhibitor GS-5806, non-nucleoside L polymerase inhibitor AZ-27, nucleoside inhibitor ALS-8112 [38], ribavirin, or palivizumab. The combination of two compounds always led to a greater inhibition than a single agent alone.

To determine whether the effect of the combination was synergistic, additive or antagonistic, the data were analyzed by the Loewe additivity model using Calcusyn software [39], which gave a Combination Index (CI) value at various effective concentrations of the compounds. A CI value of 0.9–1.1 indicates additivity, <0.9 suggests synergy, and >1.1 suggests antagonism. The combinations of EDP-938 with other RSV inhibitors showed moderate synergy. As an experimental control, adding EDP-938 to itself was additive. For details see S4 Table.

### *In vivo* efficacy in African green monkey model

**Fig 6:**
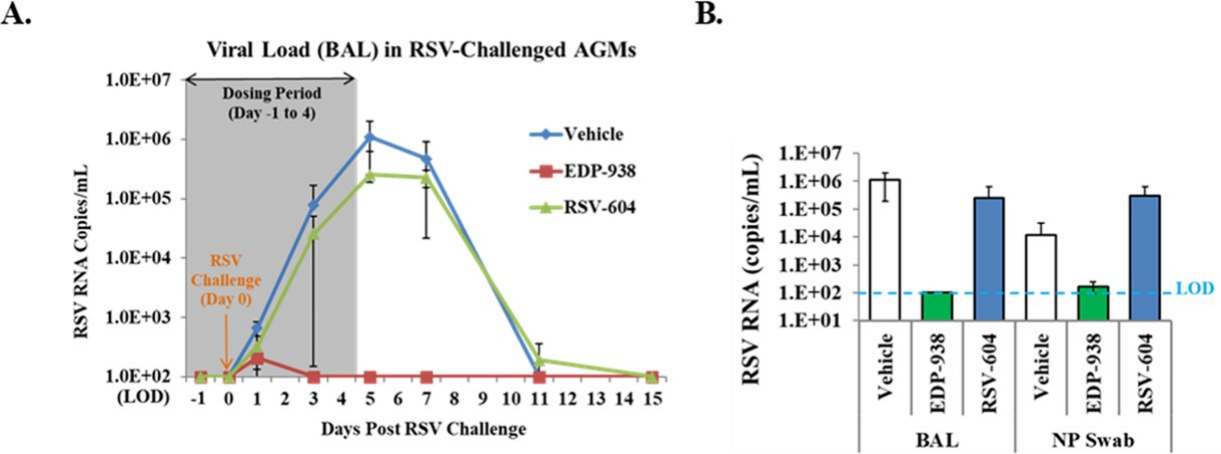
*In vivo* efficacy of EDP-938 in RSV-infected African Green monkey (AGM) model. (A) Viral load in bronchoalveolar lavage (BAL) in RSV infected AGMs. Copies of RSV RNA per mL of BAL at indicated days relative to infection challenge. EDP-938 or RSV-604 treatment period shown as shaded area. Bars represent SD. (B) Reduction in RSV RNA per mL in BAL on Day 5 or in nasopharyngeal (NP) on Day 7 post infection. LOD (limit of detection) was 100 copies/mL Bars represent SD.

African Green monkeys (AGMs) are permissive for human RSV infection and support a relatively higher level of viral replication compared to cotton rats and BALB/c mice. It has been previously validated as a non-human primate model for assessing *in vivo* efficacy of potential RSV vaccines and antiviral drugs [38]. To evaluate the activity of EDP-938 in the AGM model, 4 animals each were treated with 100 mg/kg BID with EDP-938, RSV-604, or vehicle control via oral gavage starting 24 hours prior to RSV challenge and continuing for an additional 5 days. Samples were collected through bronchoalveolar lavage (BAL) and nasopharyngeal (NP) swab at various time points to determine viral load. As shown in Fig 6A, there was robust viral replication in the RSV infected AGMs of the vehicle control group with peak viral titer of ~10^6^ copies/mL on Day 5 post infection. RSV-604 treatment did not result in significant RSV viral load reduction compared to vehicle control. In contrast, EDP-938 completely suppressed viral replication in the animals. Viral load in the NP swab samples was also determined. As shown in Fig 6B, EDP-938 treatment led to a 4-log_10_ viral load reduction to below the limit of detection (100 copies/mL) in both BAL on Day 5 and NP swab on Day 7 when the viral titer reached peak in the vehicle control group.

## Discussion

RSV is a common respiratory virus that is responsible for almost 60,000 hospitalizations among children <5 years of age in the United States [40]. In the current study, we describe the identification and characterization of novel clinical candidate, EDP-938, for the treatment of RSV. EDP-938 has nanomolar activities *in vitro* against a broad range of RSV-A and -B laboratory strains and clinical isolates. Time-of-addition studies indicate a post viral entry mechanism-of-action as EDP-938 maintains cytoprotection and RNA inhibition beyond initial infection. This contrasts with other known small molecule fusion inhibitors such as GS-5806 which are only effective prior to viral infection [41]. EDP-938’s profile more closely resembles other known replication inhibitors such as the N-protein inhibitor RSV-604, or AZ-27, ALS-8112, and PC786, each of which target the viral L protein, responsible for acting as the RNA-dependent-RNA-polymerase, capping, and methyltransferase enzyme.

The emergence of resistant viral strains on exposure to antiviral agents has the potential to impact the long-term utility of a therapeutic agent. As a result, we extensively characterized the resistance profile generated by EDP-938. Compared to resistance selection for the fusion inhibitor GS-5806 or the non-nucleoside L inhibitor AZ-27, the selection of RSV strains resistant to EDP-938 was difficult. Although were ultimately successful in the generation of resistant mutations, the level of EDP-938-resistance observed was inversely correlated with viral fitness levels. For example, the N^M109K^ mutation yielded a 67-fold EC_50_ shift, but also resulted in a loss of CPE and a 100-fold reduction in infectious virion production This resistance profile may bode well for the clinical use of EDP-938 as viral resistance appears hard to develop, and when it does such mutations carry with them significant reductions in viral fitness.

**Fig 7:**
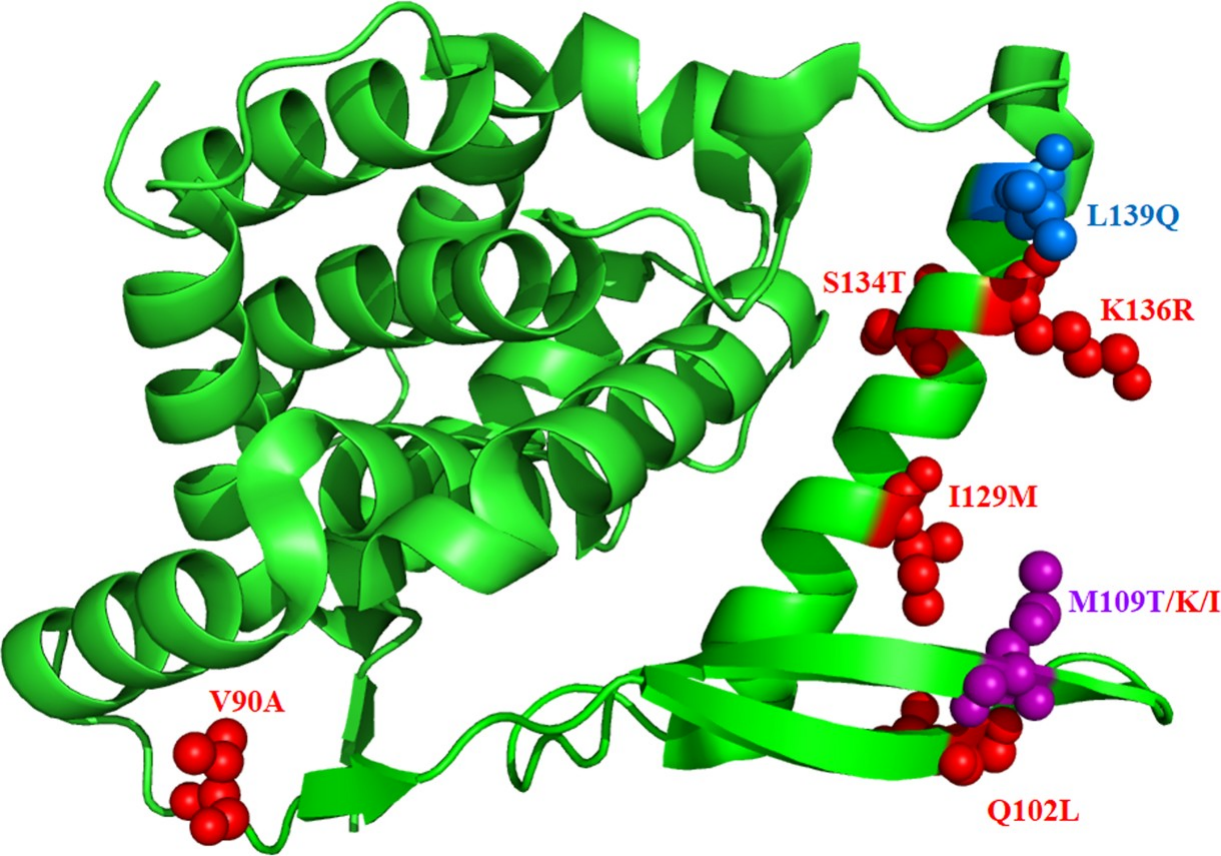
Locations of mutations mapped to RSV-A N protein. Crystal structure of amino acids 32–251. RSV-A mutations shown in red, RSV-B mutations shown in blue, purple indicates mutation seen in both. PDB file 4UC6 used for this analysis.

Upon full genome sequencing, 9 resistant variants were identified with mutations in the N and G proteins. While every resistant viral clone or population had at least 1 N mutation, only a third had G mutations. The N^M109K^ mutation granted the greatest resistance to EDP-938. Interestingly, the M109 amino acid displayed several variants, emerging multiple times across both RSV-A and -B strains. To the best of our knowledge, none of these mutations has previously been reported. Further analysis revealed that most of the EDP-938 mutations generally cluster within one ~24 angstrom solvent exposed section of the N protein, a region believed to interact with the RSV P protein (Fig 7) [42,43]. However, it seems unlikely that EDP-938 inhibits N-P interactions because its efficacy was diminished when added after 6 hpi (Fig 5), a relatively early time in the RSV replication cycle, and had no effect on either mRNA transcription or genome replication in a minigenome system. Of note, the structurally-related small-molecule inhibitor RSV-604 also generated resistant mutations within this same region of the N protein (residues 105–139) and, compared to the prototype RSV L inhibitor AZ-27, had minimal effect in a replicon assay [32]. The N protein is the most conserved of the RSV proteins, while G is the least conserved. For these reasons, we conjecture that EDP-938 is either directly targeting the N protein or is mediating its antiviral activity through the N protein via another yet unknown actor, possibly a cellular protein, given that its activity is host dependent. Further, we conjecture that EDP-938 is either inhibiting primary (but not secondary) transcription, or another very early step in the viral replication cycle. The observed G protein mutations may be acting as compensatory mutations, or perhaps are the result of random genetic drift across the ~15 passages of the virus in cell culture.

While monotherapy treatment of viral infections such as acyclovir for herpesviruses or oseltamivir for influenza exist, combining treatments targeted to distinct aspects of viral replication have often proven superior to monotherapy options. The combination potential of EDP-938 was evaluated with the either ALS-8112, AZ-27, GS-5806, ribavirin, or palivizumab. EDP-938 displayed additivity with itself and ribavirin, while presenting moderate levels of synergy with the other antiviral agents tested. Chronic viral infections may benefit more from combination therapy than acute viral infections for several reasons. With many acute infections, the host’s immune system can clear the viral pathogen if afforded the time to do so, as opposed to chronic infections where the time to mount an effective immune response is not typically a critical factor. Chronic conditions often go untreated for longer periods as compared to acute ailments, enabling chronic infections more time to diversify their genetic pool through selection and genetic drift. Additionally, chronic conditions often necessitate prolonged treatment courses as compared to acute infections, allowing the chronic viral pathogen more time to develop resistance mutations, something which combination therapy could mitigate against. While RSV is generally considered an acute infection, it has yet to be determined whether monotherapy or combination treatment of RSV will be required to effectively repress viral replication in a clinical setting.

The African green monkey model was selected for EDP-938 *in vivo* studies for its higher level of viral replication versus cotton rats and BALB/c mice. RSV-604, a compound whose mechanism of action likely matches that of EDP-938 has previously been shown to have an antiviral effect in a phase 2 study of RSV-infected patients following stem cell transplantation. In this previous trial, patients who achieved RSV-604 levels in excess of the EC_90_ experienced a 2.31 log reduction in viral load [14]. In the African Green monkey study presented here, EDP-938 displayed an ~4-log_10_ reduction in peak viral load, while RSV-604 was unable to reduce viral load in a statistically significant manner. Additionally, following the cessation of treatment on day 4-post infection, no viral rebound was observed in the BAL fluid above the limit of detection. The lack of antiviral activity observed with RSV-604 could be a function of relatively poor potency compared to EDP-938 (Fig 1) and/or insufficient tissue exposure. While in this study the animals were not sacrificed and thus the drug level in the lung could not be determined, plasma samples were taken at 12h after the last dose (C_trough_) to confirm drug exposure. EDP-938 had a mean plasma C_trough_ of 1479 nM, which was ~15x of its EC_90_. In contrast, RSV-604, given at the same dose, had a mean plasma C_trough_ of 637 nM, which was well below its EC_90_. This was likely the reason why EDP-938 worked in the model while RSV-604 did not. It is reasonable to project that given the same dose or exposure EDP-938 should perform better than RSV-604 in RSV infected patients as well.

In summary, EDP-938 is a potent nanomolar inhibitor of all assayed RSV strains *in vitro*. An *in vitro* selectivity index of over 2,380 suggests an excellent safety window. It displays a high barrier to resistance as compared to compounds targeting fusion or polymerase proteins. Mutation mapping suggests a mechanism of action mediated by the N protein. Resistance mutations which do arise appear to come at a severe fitness cost to the virus, thus minimizing concerns of the widespread emergence of drug resistant strains. EDP-938 displays additive to synergistic effects when given in combination with other anti-RSV compounds and treatments which suggests a potential for combination therapy options. Clinical studies evaluating the effects of EDP-938 in patients with RSV infection are currently ongoing.

## Materials and methods

### Ethics *s*tatement

The African Green Monkeys (Chlorocebus), used in this study were housed at BIOQUAL, Inc., in accordance with the recommendations of the Association for Assessment and Accreditation of Laboratory Animal Care International Standards and with the recommendations in the Guide for the Care and Use of Laboratory Animals of the United States—National Institutes of Health. The Institutional Animal Use and Care Committee of BIOQUAL approved this experiment (protocol no. 16–105). When immobilization was necessary, the animals were sedated by intramuscularly injection with 10 mg/kg of Ketamine HCl. All efforts were made to minimize suffering. Details of animal welfare and steps taken to ameliorate suffering were in accordance with the recommendations of the Weatherall report, "The use of non-human primates in research." Animals were housed in an air-conditioned facility with an ambient temperature of 21–25°C, a relative humidity of 40%-60% and a 12 h light/dark cycle. Animals were socially housed when possible or individually housed if no compatible pairing could be found. Animals also received appropriate environmental enrichment. The animals were housed in suspended stainless steel wire-bottomed cages during the quarantine period and in HEPA filtered microisolator caging during the RSV challenge phase, to minimize virus transmission. The animals were provided with a commercial primate diet and fresh fruit twice daily, with water freely available at all times. RSV infected AGMs were humanely euthanized in accordance with the American Veterinary Medical Association Guidelines on Euthanasia, 2020.

### *In vitro* compound screen against RSV laboratory strains

HEp-2, Vero, and BHK were seeded at 8,000 cells per well (cpw) in growth medium (Dulbecco’s Modified Eagle Medium (DMEM) with 1% GlutaMAX, 1% Penicillin/Streptomycin (P/S), 1% Non-Essential Amino Acids (NEAA), 10% Fetal Bovine Serum (FBS)) or (Minimum Essential Medium (MEM) with 10% FBS, 1% NEAA, 1% P/S) or (Eagle’s Minimum Essential Medium with L-Glutamine, with 10% FBS, 1% P/S) respectively. A549 seeded at 3,000 cpw in F-12K Kaighn’s Modification nutrient mixture with 10% FBS, 1% NEAA, 1% P/S. Cells seeded at time of infection in 96-well plates, final well volume 100μL. Compounds suspended in DMSO, serially diluted and added to wells at 0.5% final DMSO concentration. Viral stocks stabilized with 25% sucrose, previously titered using plaque assay, were added at desired MOI. Sucrose added to cells-only control wells to match percent added with virus. Unless otherwise noted, RSV-A strains were tested at 0.1 MOI and incubated for 5 days in a 37°C, 5% CO_2_ humidified incubator, and RSV-B at 0.5 MOI for 6 days. Cytopathic effect (CPE) determined using ATPlite (Perkin Elmer) measured with Envision luminometer. For RT-qPCR, RNA was extracted from whole well using Ambion RNAqueous-96 Automated Kit, and RT-qPCR run using Applied Biosystems TaqMan RNA-to-CT 1-Step Kit. Primer/probes set for N gene (RSV-A: for: CATCCAGCAAATACACCATCCA, rev: TTCTGCACATCATAATTAGGA-GTATCAA, probe: FAM-CGGAGCACAGGAGAT-NFQ-MGB; RSV-B: for: CTGTCATCCAGCAAATACACT-ATTCA, rev: GCACATCATAATTGGGAGTGTCA, probe: FAM-CGTAGTACAGGAGATAAT-NFQ-MGB) run with 1kb N gene standard of known concentration. All EC_50_s were calculated using the XLFit four-parameter logistic model 200 in Microsoft Excel.

### *In vitro* compound screen against RSV clinical isolate strains

RSV clinical isolates were obtained from Baylor University and Medical College of Wisconsin. MOI of 0.1 used for all clinical isolates except RSV-A 629-9-2 (0.01) and RSV-B 629-24/2007 (0.0044) due to low titer. Virospot reduction assay was run by ViroClinics (Rotterdam, the Netherlands) against 20 additional RSV clinical isolates [44,45].

### Activity of EDP-938 in Primary Human Bronchial Epithelial Cells (HBECs), Primary Cotton Rat Lung Cells, and Primary Neonatal Lamb Bronchial Epithelial Cells

Primary undifferentiated HBECs obtained from ATCC or PromoCell. Maintained in ATCC Airway Epithelial Cell Basal Medium with HLL Supplement, L-glutamine, Extract P, Airway Epithelial Cell Supplement or PromoCell Airway Epithelial Cell Growth Medium with Supplement Mix and 1% P/S respectively. Collagen-coated plates seeded with 8,000 cpw and infected at 0.1 MOI. Incubated 6 or 7 days with RSV-A and -B strains respectively. Primary cotton rat lung cells were harvested and maintained in Minimum Essential Medium with 2mM L-Glutamine, Earle’s Balanced Salts, 30 μg/mL Gentamicin, 2% P/S, 2% Amphotericin B. Assay was run using Minimum Essential Medium with 2mM L-Glutamine, Earle’s Balanced Salts, 10% FBS, 1% P/S, 1% NEAA, 10 ng/mL hEGF, 1x Lactalbumin Hydrolysate. Collagen-coated plates seeded with 8,000 cpw and infected at 0.1 MOI. Incubated 5 days with RSV-A strain. Primary neonatal lamb bronchial epithelial cells were harvested and maintained in Bronchial Epithelial Cell (BEC) Growth Medium. Seeded at 10,000 cpw in BEC medium, then washed twice with SF-DMEM the next day. Infected at 0.1 MOI, incubated 6 days with RSV-A strain.

### Cytotoxicity

HEp-2, A549, and CHO cells were seeded at 3,000 cpw in their respective growth medium. CHO growth medium used was RPMI medium 1640 with 10% FBS, 1% P/S, 1% GlutaMAX. HEK-293 cells seeded at 10,000 cpw in DMEM growth medium. Undifferentiated HBECs were seeded in collagen-coated 96-well plates at 7,500 cpw in appropriate growth medium. Compound added and incubated for 3 days (4 days for HBECs) as described above. Plates read with ATPlite and CC_50_ calculated as EC_50_s as described above. SI = CC_50_/EC_50_ within a cell line.

### Viral specificity

EDP-938 were tested against human metapneumovirus and parainfluenza virus 3 using PIV-3_JS_GFP and hMPV-Can97-83_GFP (ViraTree) in LLCMK2 cells (ATCC, cat# CCL-7) at Katholieke Universiteit Leuven. The activities against human rhinovirus 14 (1059 strain), MERS coronavirus (EMC strain), influenza A (H1N1) and B (Brisbane/60/2008) viruses were determined in Hela-Ohio, Vero 76, and MDCK cells at NIAID/Utah State University. Another human coronavirus, HCoV-229E, was assayed in MRC-5 cells at Imquest Biosciences, Inc. (Frederick, Maryland). The activity against Measles virus Edmonston strain was evaluated in MRC-5 cells at Southern Research Institute (Frederick, Maryland). Hepatitis B virus (HBV) and hepatitis C virus (HCV) activities were evaluated at Enanta Pharmaceuticals Inc. using HepAD38 cells and a subgenomic genotype 1b HCV replicon kindly provided by Dr. Christoph Seeger (Fox Chase Cancer Center) and Dr. Ralf Bartenschlager (Heidelberg University), respectively.

### Time-of-addition

As above, but 8,000 HEp-2 cpw seeded 1-day pre-infection. 2h pre-infection compound or media added. Plates incubated 2h. Viral infection at 0.1 MOI, incubated 1h. Viral inoculum removed, cells washed, and media replaced with compound for -2h sample. Compound added at 2h, 6h, and 24h post-infection to appropriate plates. 5dpi cell viability calculated as described above.

For single round infection synchronized infection 25,000 HEp-2 cpw seeded 1-day pre-infection. Plates brought to 4°C, viral infection at 3 MOI calculated for 30,000 cpw incubated at 4°C 1hr. Compound added to 0h plate and plates moved to 37°C. Compound added at 2h, 4h, 6h, and 8h post-incubation (when plates moved to 37°C) to appropriate plates. 24 hpi viral copies measured with RT-qPCR as described above.

### Synchronized infection and analysis of primary viral transcription

To synchronize infection, HEp-2 cells seeded with or without coverslips, were chilled on ice for ~ 5 minutes, washed twice with ice-cold serum free Opti-MEM and infected with a chilled RSV A2 inoculum at an MOI of 3 in Opti-MEM containing 2% FBS. The virus was allowed to adsorb for 2 h at +4°C (without disturbance), after which at 0 hpi the inoculum was supplemented with 37°C pre-warmed media (Opti-MEM containing 2% FBS) containing DMSO or 1000 nM EDP-938 (diluted in DMSO, such that the concentration of DMSO was equivalent in each well) and incubated at 37°C. For immunofluorescence microscopy analyses, at 8 hpi cells seeded on coverslips were fixed with 5% formaldehyde, 2% sucrose in phosphate buffered saline for 30 min, permeabilized with 0.5% Igepal, 10% sucrose in phosphate buffered saline for 20 min, and incubated with an antibody toward RSV N protein (Serotec). Following washing in phosphate buffered saline, cells were incubated with isotype specific secondary antibody, labelled with Alexafluor 588 and DAPI. Cells were analyzed by fluorescence microscopy. For RT-qPCR analyses, cells were harvested at the indicated times and RNA extracted using Trizol (ThermoFisher) according to manufacturer’s instructions, except that following isopropanol precipitation it was resuspended in 2 M NTE (2 M NaCl, 40 mM Tris [pH 7.4], 1 mM EDTA), extracted with phenol and chloroform, and precipitated with ethanol. RNA pellets were resuspended in 50 μl of RNase free water. 500 ng of RNA was annealed simultaneously to a GAPDH primer and N specific primer to detect positive sense RNA and reverse transcribed using superscript IV (Thermofisher) according to manufacturer’s instructions. 2 μl of cDNA was then subjected to qPCR. Amplicons were detected using iTaq Universal Sybr Green Supermix (Bio-Rad), and quantified using the ΔΔCt method relative to the mean expression of housekeeping gene GAPDH. Primer sets for N gene (for- ATGGGAGAGGTAGCTCCAGA, rev- AGCTCTCCTAATCACGGCTG) and GAPDH (for- GTCGGAGTCAACGGATT, rev- AAGCTTCCCGTTCTCAG). The data presented represent one of two independent experiments, performed with slightly different timepoints. In each experiment, the reverse transcription step was performed twice and each set of cDNA was analyzed in duplicate by qPCR.

### *In vitro* resistance study

#### RSV drug resistance selection

Both T25 flasks and 24-well plates were utilized to generate drug resistant mutants (DR/DRMs). Cells (HEp-2 for RSV-A Long, and A549 for RSV-B VR-955) were seeded well below confluency. Viral infection initiated at 0.05–0.1 MOI. EDP-938 initially administered at 1X, 4X, 10X, 8X, and 50X EC_50_ anywhere from 3–10 times each. GS-5806 and AZ-27 initiated at 10X and 50X EC_50_ 1 time each. Passaged 1 DMSO-only culture to track DMSO / random genetic drift. All samples had 0.1% final DMSO concentration. Collected upon first of ~70% CPE or 7dpi for RSV-A. RSV-B lost CPE effect after several passages and was monitored by RT-qPCR. Virus was collected by scraping cells into media, adding 25% sucrose, using LN2 for 2x snap freezes of pellet mixing in supernatant between each freeze. Passaging was done with 10–100μL of viral stock. Stocks were passaged at 0.5X, 1X, and 2X EDP-938 from the previous passage each round. Loss of virus resulted in backtracking or passage of a lower concentration stock. Successful growth resulted in discard of active lower concentration stocks. Virus stored long term at -80°C. Each RSV-A DR population was plaque purified using 0.3% agarose overlay in growth media (3 plaques picked each) and amplified by passage with the top respective concentration of EDP-938.

#### Sanger sequencing

RNA extracted as above. RT-PCR used SuperScript One Step Long Kit with primers covering the entire RSV genome in sections. Gel purified product sent to Quintara Biosciences for Sanger sequencing. Chromatograms from DMSO/WT results compared to DRMs in house.

#### Characterization of RSV drug-resistant strains

RSV-A DR strains titered by plaque assay as previously described [46]. CPE and RT-qPCR compound testing performed as described above at a MOI of 0.1. DMSO passaged virus used as WT control. EDP-938, RSV-604, and GS-5806 tested against each strain. Fold shift = DRM EC_50_ / WT EC_50_.

Viral fitness performed as CPE assay above with compound omitted, 8,000 HEp-2 cpw, 0.01 MOI, and 6dpi endpoint. Inoculum used for “day 0” pfu value. CPE quantified as above on days 0–6. Pfu samples collected days 1–6 by adding equal volume media +50% sucrose, scraping cell debris and collecting entire well as above. RSV-B DRMs infected using RT-qPCR-determined genomic equivalence with RT-qPCR readout as above vs. DMSO passaged WT strain.

### RSV reverse genetics system

Preparation and recovery of recombinant WT and mutant RSV was performed using the bacterial artificial chromosome (BAC)-based system described in Stobart et al [47]. Mutagenesis performed on a BAC containing the antigenome of RSV A2-line19F (pSynk-AZ) using an Agilent site-directed mutagenesis kit following manufacturer’s instructions. Upon mutation confirmation, WT or modified versions of pSynk-AZ along with the four helper plasmids and a plasmid encoding T7 polymerase were transfected into Vero cells. Cells incubated at 37°C. Replaced culture media every 2–3 days until detection of mKATE2 fluorescent foci indicative of active recombinant RSV propagation. Cells expanded and incubated at 37°C until mKATE2 fluorescence detection in >75% of a T25 flask. Working viral stocks were expanded in HEp2 cells. Titration performed by a modified plaque assay in HEp2 cells, assessing the number of fluorescent foci 7 dpi. Mutations confirmed by Sanger sequencing as described previously.

### *In vitro* combination study

As described above with 8,000 HEp-2 cpw, 0.1 MOI RSV-A Long, 5-day endpoint, and CPE readout. Compounds added in serial 1.3-fold 6-point dilutions across both X- and Y-axes of plate. Plates run in quadruplicate with one uninfected plate for cytotoxicity. Combination indexes calculated using Loewe additivity model using CalcuSyn software from Biosoft.

### African green monkey model

A study of EDP-938 and RSV-604 in the African Green monkey (AGM) animal model was performed by BIOQUAL, Inc (Rockville, Maryland). Methods were similar to those run in a study by Ispas et al. [48]. Six (6) male and 6 female AGMs (*Chlorocebus*) of 4–5 kg were purchased from PrimGen (Hines, Illinois). Each animal was challenged with 2 x 10^5^ PFU of RSV A2 viral inoculum (Virapur, San Diego, CA). Four animals (2 per sex) in each group were treated with EDP-938, RSV-604, or vehicle control (0.5% w/v methylcellulose). The animals were dosed via oral gavage twice daily (every 12 hours), starting 24 hours prior to RSV challenge, and then continuing for an additional 5 days (6 total days of dose administration). Bronchoalveolar lavage (BAL) samples and nasopharyngeal (NP) swabs were collected for viral load determination.

## Supporting information

S1 Table *In vitro* cytotoxicity of EDP-938. (TIF)

S2 Table *In vitro* activity of EDP-938 against other viruses. (TIF)

S3 Table Fitness of resistance mutant vs. wild-type (WT) virus. (TIF)

S4 Table Analysis of the effect of combination of EDP-938 with other RSV inhibitors. (TIF)

S1 Fig Passage history of *in vitro* selection of RSV resistant strains. Viral passaging in the presence of increasing concentrations of compound. In each Panel, grayscale curves represent stocks unable to survive with black markers denoting the terminal passage. Resistant strains are indicated by colored lines. (A) RSV-A Long passaged in the presence of EDP-938. 7 attempts to grow virus at 4x and 8x the EDP-938 EC_50_ value resulted in immediate loss of virus. EDP-938 increases were attempted every passage; all failures but terminal omitted from graph. (B) RSV-B VR-955 passaged. 20 separate attempts resulted in total loss of virus. Some curves slightly offset to display better. (C) GS-5806- and (D) AZ-27-induced viral resistance. Compound increases were not attempted every passage.
(TIF)

## References

[1] Nair H, Simoes EA, Rudan I, Gessner BD, Azziz-Baumgartner E, Zhang JSF, et al. Global and regional burden of hospital admissions for severe acute lower respiratory infections in young children in 2010: a systematic analysis. Lancet. 2013;381(9875):1380–90. 10.1016/S0140-6736(12)61901-1 23369797 PMC3986472

[2] Falsey AR, Hennessey PA, Formica MA, Cox C, Walsh EE. Respiratory syncytial virus infection in elderly and high-risk adults. N Engl J Med. 2005;352(17):1749–59. 10.1056/NEJMoa043951 . 15858184

[3] Chatzis O, Darbre S, Pasquier J, Meylan P, Manuel O, Aubert JD, et al. Burden of severe RSV disease among immunocompromised children and adults: a 10 year retrospective study. BMC Infect Dis. 2018;18(1):111. 10.1186/s12879-018-3002-3 29510663 PMC5838875

[4] Shi T, McAllister DA, O’Brien KL, Simoes EAF, Madhi SA, Gessner BD, et al. Global, regional, and national disease burden estimates of acute lower respiratory infections due to respiratory syncytial virus in young children in 2015: a systematic review and modelling study. Lancet. 2017;390(10098):946–58. 10.1016/S0140-6736(17)30938-8 28689664 PMC5592248

[5] Stockman LJ, Curns AT, Anderson LJ, Fischer-Langley G. Respiratory syncytial virus-associated hospitalizations among infants and young children in the United States, 1997–2006. Pediatr Infect Dis J. 2012;31(1):5–9. 10.1097/INF.0b013e31822e68e6 . 21817948

[6] Scheltema NM, Gentile A, Lucion F, Nokes DJ, Munywoki PK, Madhi SA, et al. Global respiratory syncytial virus-associated mortality in young children (RSV GOLD): a retrospective case series. Lancet Glob Health. 2017;5(10):e984–e91. 10.1016/S2214-109X(17)30344-3 28911764 PMC5599304

[7] Loustaud-Ratti V, Debette-Gratien M, Jacques J, Alain S, Marquet P, Sautereau D, et al. Ribavirin: Past, present and future. World J Hepatol. 2016;8(2):123–30. Epub 2016/01/26. 10.4254/wjh.v8.i2.123 26807208 PMC4716528

[8] Burrows FS, Carlos LM, Benzimra M, Marriott DJ, Havryk AP, Plit ML, et al. Oral ribavirin for respiratory syncytial virus infection after lung transplantation: Efficacy and cost-efficiency. J Heart Lung Transplant. 2015;34(7):958–62. Epub 2015/03/11. 10.1016/j.healun.2015.01.009 . 25753833

[9] Lewinsohn DM, Bowden RA, Mattson D, Crawford SW. Phase I study of intravenous ribavirin treatment of respiratory syncytial virus pneumonia after marrow transplantation. Antimicrob Agents Chemother. 1996;40(11):2555–7. Epub 1996/11/01. 10.1128/AAC.40.11.2555 8913463 PMC163574

[10] Geskey JM, Thomas NJ, Brummel GL. Palivizumab: a review of its use in the protection of high risk infants against respiratory syncytial virus (RSV). Biologics. 2007;1(1):33–43. 19707346 PMC2721348

[11] Collins PL, Fearns R, Graham BS. Respiratory syncytial virus: virology, reverse genetics, and pathogenesis of disease. Curr Top Microbiol Immunol. 2013;372:3–38. 10.1007/978-3-642-38919-1_1 24362682 PMC4794264

[12] Mackman RL, Sangi M, Sperandio D, Parrish JP, Eisenberg E, Perron M, et al. Discovery of an oral respiratory syncytial virus (RSV) fusion inhibitor (GS-5806) and clinical proof of concept in a human RSV challenge study. J Med Chem. 2015;58(4):1630–43. 10.1021/jm5017768 . 25574686

[13] Chemaly RF, Dadwal SS, Bergeron A, Ljungman P, Kim YJ, Cheng GS, et al. A phase 2, randomized, double-blind, placebo-controlled trial of presatovir for the treatment of respiratory syncytial virus upper respiratory tract infection in hematopoietic-cell transplant recipients. Clin Infect Dis. 2019. 10.1093/cid/ciz1166 . 31793991 PMC7108134

[14] Marty FM, Chemaly RF, Mullane KM, Lee DG, Hirsch HH, Small CB, et al. A Phase 2b, Randomized, Double-blind, Placebo-Controlled Multicenter Study Evaluating Antiviral Effects, Pharmacokinetics, Safety, and Tolerability of Presatovir in Hematopoietic Cell Transplant Recipients with Respiratory Syncytial Virus (RSV) Infection of the Lower Respiratory Tract. Clin Infect Dis. 2019. 10.1093/cid/ciz1167 . 31915807 PMC7108198

[15] Hanfelt-Goade D. A Phase 2b, Randomized, Double-Blind, Placebo-Controlled Trial of Presatovir (GS-5806), a Novel Oral RSV Fusion Inhibitor, for the Treatment of Respiratory Syncytial Virus (RSV) in Hospitalized Adults. NEW INSIGHTS IN ACUTE PULMONARY INFECTIONS.A4457-A

[16] Stray K, Perron M, Porter DP, Anderson F, Lewis SA, Perry J, et al. Drug resistance assessment following administration of RSV fusion inhibitor presatovir to participants experimentally infected with respiratory syncytial virus. J Infect Dis. 2020. 10.1093/infdis/jiaa028 . 31971597

[17] Porter DP, Guo Y, Perry J, Gossage DL, Watkins TR, Chien JW, et al. Assessment of drug resistance during phase 2b clinical trials of presatovir in adults naturally infected with respiratory syncytial virus. Antimicrob Agents Chemother. 2020. 10.1128/AAC.02312-19 . 32071058 PMC7449164

[18] Stevens M, Rusch S, DeVincenzo J, Kim YI, Harrison L, Meals EA, et al. Antiviral Activity of Oral JNJ-53718678 in Healthy Adult Volunteers Challenged With Respiratory Syncytial Virus: A Placebo-Controlled Study. J Infect Dis. 2018;218(5):748–56. 10.1093/infdis/jiy227 . 29684148

[19] Zheng X, Gao L, Wang L, Liang C, Wang B, Liu Y, et al. Discovery of Ziresovir as a Potent, Selective, and Orally Bioavailable Respiratory Syncytial Virus Fusion Protein Inhibitor. J Med Chem. 2019;62(13):6003–14. 10.1021/acs.jmedchem.9b00654 . 31194544

[20] DeVincenzo J, Tait D, Efthimiou J, Mori J, Kim YI, Thomas E, et al. A Randomized, Placebo-Controlled, Respiratory Syncytial Virus Human Challenge Study of the Antiviral Efficacy, Safety, and Pharmacokinetics of RV521, an Inhibitor of the RSV-F Protein. Antimicrob Agents Chemother. 2020;64(2). 10.1128/AAC.01884-19 31712214 PMC6985722

[21] Wang G, Deval J, Hong J, Dyatkina N, Prhavc M, Taylor J, et al. Discovery of 4’-chloromethyl-2’-deoxy-3’,5’-di-O-isobutyryl-2’-fluorocytidine (ALS-8176), a first-in-class RSV polymerase inhibitor for treatment of human respiratory syncytial virus infection. J Med Chem. 2015;58(4):1862–78. 10.1021/jm5017279 . 25667954

[22] Xiong H, Foulk M, Aschenbrenner L, Fan J, Tiong-Yip CL, Johnson KD, et al. Discovery of a potent respiratory syncytial virus RNA polymerase inhibitor. Bioorg Med Chem Lett. 2013;23(24):6789–93. 10.1016/j.bmcl.2013.10.018 . 24211022

[23] Coates M, Brookes D, Allen H, Fordyce E, Colley T, Hunt F, et al. Preclinical characterization of PC786, a potent antiviral inhibitor of respiratory syncytial virus replication. XVIII International Symposium on Respiratory Viral Infections; March 31 –April 2, 2016; Lisbon, Portugal2016.

[24] Beigel JH, Nam HH, Adams PL, Krafft A, Ince WL, El-Kamary SS, et al. Advances in respiratory virus therapeutics—A meeting report from the 6th isirv Antiviral Group conference. Antiviral Res. 2019;167:45–67. 10.1016/j.antiviral.2019.04.006 30974127 PMC7132446

[25] DeVincenzo JP, McClure MW, Symons JA, Fathi H, Westland C, Chanda S, et al. Activity of oral als-008176 in a respiratory syncytial virus challenge study. N Engl J Med. 2015;373(21):2048–58. 10.1056/NEJMoa1413275 . 26580997

[26] SEC filing 2019. Available from: https://www.sec.gov/Archives/edgar/data/200406/000020040619000016/a20190321alios8-k.htm.

[27] Respiratory syncytial virus immune globulin intravenous: indications for use. American Academy of Pediatrics Committee on Infectious Diseases, Committee on Fetus and Newborn. Pediatrics. 1997;99(4):645–50. . 9093323

[28] Carter MC, Alber DG, Baxter RC, Bithell SK, Budworth J, Chubb A, et al. 1,4-Benzodiazepines as inhibitors of respiratory syncytial virus. J Med Chem. 2006;49(7):2311–9. 10.1021/jm051185t . 16570927

[29] Chapman J, Abbott E, Alber DG, Baxter RC, Bithell SK, Henderson EA, et al. RSV604, a novel inhibitor of respiratory syncytial virus replication. Antimicrob Agents Chemother. 2007;51(9):3346–53. 10.1128/AAC.00211-07 17576833 PMC2043207

[30] Chapman J, Cockerill GS. Discovery and development of RSV604. In: Kazmierski WM, editor. Antiviral Drugs: From Basic Discovery Through Clinical Trials, First Edition: John Wiley & Sons, Inc.; 2011

[31] Shook BC, Kim IJ, Blaisdell TP, Yu J, Panarese J, Or YS. Benzodiazepine derivatives as RSV inhibitors. WO2017015449. 2017.

[32] Challa S, Scott AD, Yuzhakov O, Zhou Y, Tiong-Yip CL, Gao N, et al. Mechanism of action for respiratory syncytial virus inhibitor RSV604. Antimicrob Agents Chemother. 2015;59(2):1080–7. Epub 2014/12/03. 10.1128/AAC.04119-14 25451060 PMC4335855

[33] Tiong-Yip CL, Aschenbrenner L, Johnson KD, McLaughlin RE, Fan J, Challa S, et al. Characterization of a respiratory syncytial virus L protein inhibitor. Antimicrob Agents Chemother. 2014;58(7):3867–73. 10.1128/AAC.02540-14 24777090 PMC4068518

[34] Rincheval V, Lelek M, Gault E, Bouillier C, Sitterlin D, Blouquit-Laye S, et al. Functional organization of cytoplasmic inclusion bodies in cells infected by respiratory syncytial virus. Nat Commun. 2017;8(1):563. 10.1038/s41467-017-00655-9 28916773 PMC5601476

[35] Blanchard EL, Braun MR, Lifland AW, Ludeke B, Noton SL, Vanover D, et al. Polymerase-tagged respiratory syncytial virus reveals a dynamic rearrangement of the ribonucleocapsid complex during infection. PLoS Pathog. 2020;16(10):e1008987. Epub 2020/10/09. 10.1371/journal.ppat.1008987 33031461 PMC7575074

[36] Garcia J, Garcia-Barreno B, Vivo A, Melero JA. Cytoplasmic inclusions of respiratory syncytial virus-infected cells: formation of inclusion bodies in transfected cells that coexpress the nucleoprotein, the phosphoprotein, and the 22K protein. Virology. 1993;195(1):243–7. Epub 1993/07/01. 10.1006/viro.1993.1366 . 8317099

[37] Galloux M, Risso-Ballester J, Richard CA, Fix J, Rameix-Welti MA, Eleouet JF. Minimal Elements Required for the Formation of Respiratory Syncytial Virus Cytoplasmic Inclusion Bodies In Vivo and In Vitro. mBio. 2020;11(5). Epub 2020/09/24. 10.1128/mBio.01202-20 32963000 PMC7512546

[38] Deval J, Hong J, Wang G, Taylor J, Smith LK, Fung A, et al. Molecular basis for the selective inhibition of respiratory syncytial virus rna polymerase by 2’-fluoro-4’-chloromethyl-cytidine triphosphate. PLoS Pathog. 2015;11(6):e1004995. 10.1371/journal.ppat.1004995 26098424 PMC4476725

[39] Chou TC, Talalay P. Quantitative analysis of dose-effect relationships: the combined effects of multiple drugs or enzyme inhibitors. Adv Enzyme Regul. 1984;22:27–55. 10.1016/0065-2571(84)90007-4 . 6382953

[40] CDC report 2019. Available from: https://wwwn.cdc.gov/nndss/conditions/respiratory-syncytial-virus-associated-mortality/case-definition/2019/.

[41] Brookes DW, Coates M, Allen H, Daly L, Constant S, Huang S, et al. Late therapeutic intervention with a respiratory syncytial virus L-protein polymerase inhibitor, PC786, on respiratory syncytial virus infection in human airway epithelium. Br J Pharmacol. 2018;175(12):2520–34. Epub 2018/03/27. 10.1111/bph.14221 29579332 PMC5980447

[42] Ouizougun-Oubari M, Pereira N, Tarus B, Galloux M, Lassoued S, Fix J, et al. A Druggable Pocket at the Nucleocapsid/Phosphoprotein Interaction Site of Human Respiratory Syncytial Virus. J Virol. 2015;89(21):11129–43. 10.1128/JVI.01612-15 26246564 PMC4621127

[43] Galloux M, Tarus B, Blazevic I, Fix J, Duquerroy S, Eleouet JF. Characterization of a viral phosphoprotein binding site on the surface of the respiratory syncytial nucleoprotein. J Virol. 2012;86(16):8375–87. Epub 2012/05/25. 10.1128/JVI.00058-12 22623798 PMC3421704

[44] van Baalen CA, Jeeninga RE, Penders GH, van Gent B, van Beek R, Koopmans MP, et al. ViroSpot microneutralization assay for antigenic characterization of human influenza viruses. Vaccine. 2017;35(1):46–52. Epub 2016/12/03. 10.1016/j.vaccine.2016.11.060 . 27899226

[45] Baalen CAv, Keawcharoen J, Waal Ld, Penders G, Roelofse T, Nirmala E, et al. Influenza virus and RSV ViroSpot™ Assays for high-throughput virology testing. In: biosciences V, editor.: Viroclinics biosciences; 2020. p. 1.

[46] McKimm-Breschkin JL. A simplified plaque assay for respiratory syncytial virus—direct visualization of plaques without immunostaining. J Virol Methods. 2004;120(1):113–7. Epub 2004/07/06. 10.1016/j.jviromet.2004.02.020 . 15234816

[47] Stobart CC, Hotard AL, Meng J, Moore ML. BAC-Based Recovery of Recombinant Respiratory Syncytial Virus (RSV). Methods Mol Biol. 2017;1602:111–24. 10.1007/978-1-4939-6964-7_8 . 28508217

[48] Ispas G, Koul A, Verbeeck J, Sheehan J, Sanders-Beer B, Roymans D, et al. Antiviral activity of tmc353121, a respiratory syncytial virus (rsv) fusion inhibitor, in a non-human primate model. PLoS One. 2015;10(5):e0126959. 10.1371/journal.pone.0126959 26010881 PMC4444337

