# Supplementary figures and images for: EDP-938, a novel nucleoprotein inhibitor of respiratory syncytial virus, demonstrates potent antiviral activities in vitro and in a non-human primate model
Source: PLoS Pathog. 2021 Mar 15;17(3):e1009428. doi: 10.1371/journal.ppat.1009428 (PMC7993833; doi:10.1371/journal.ppat.1009428)

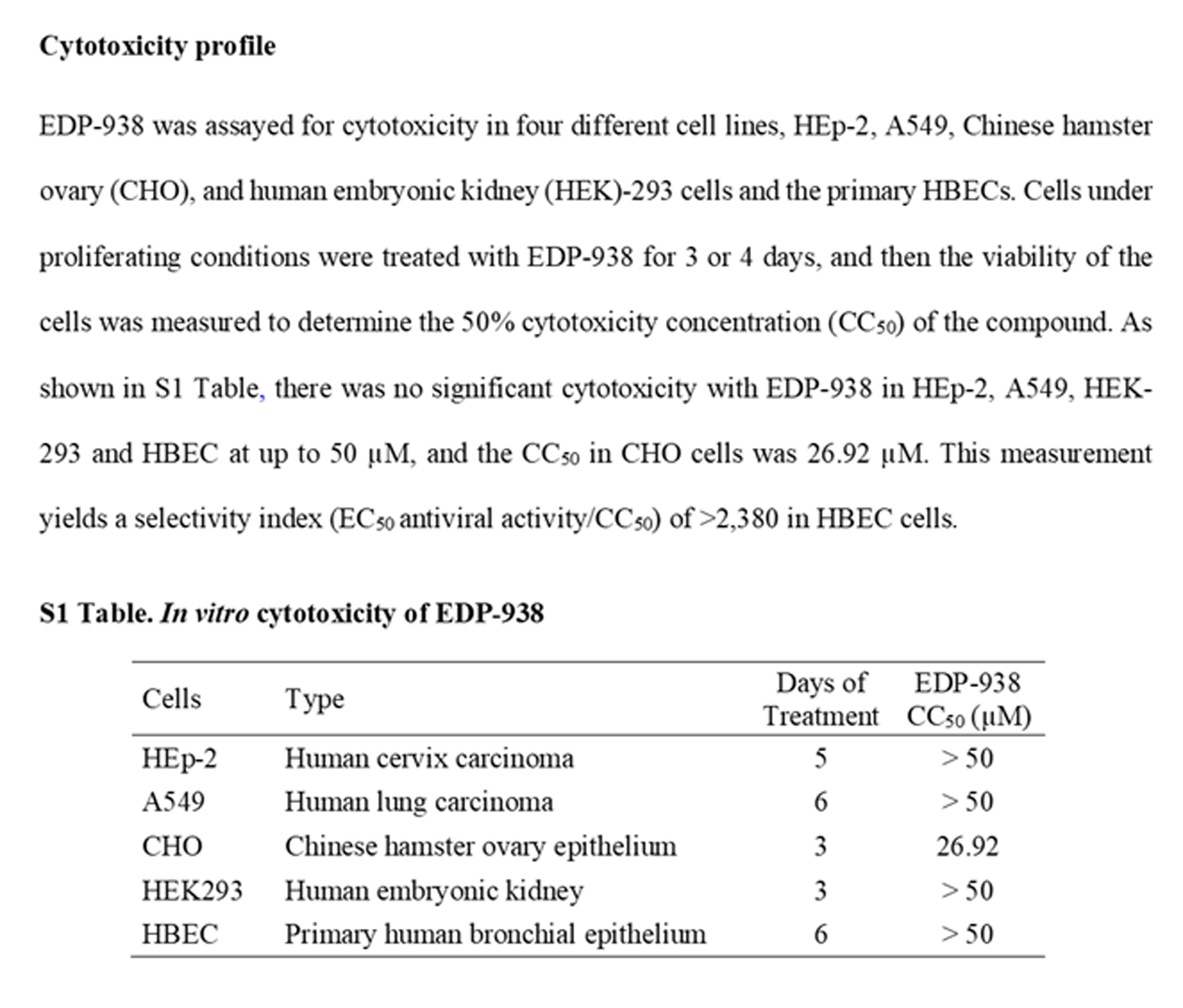

Supplement: S1 Table — (TIF) [file ppat.1009428.s001.tif]

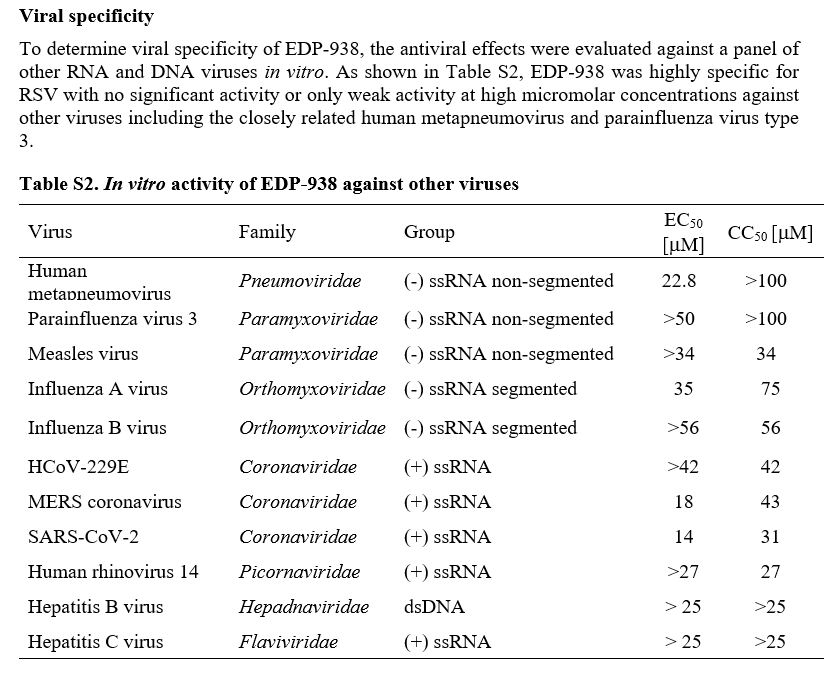

Supplement: S2 Table — (TIF) [file ppat.1009428.s002.tif]

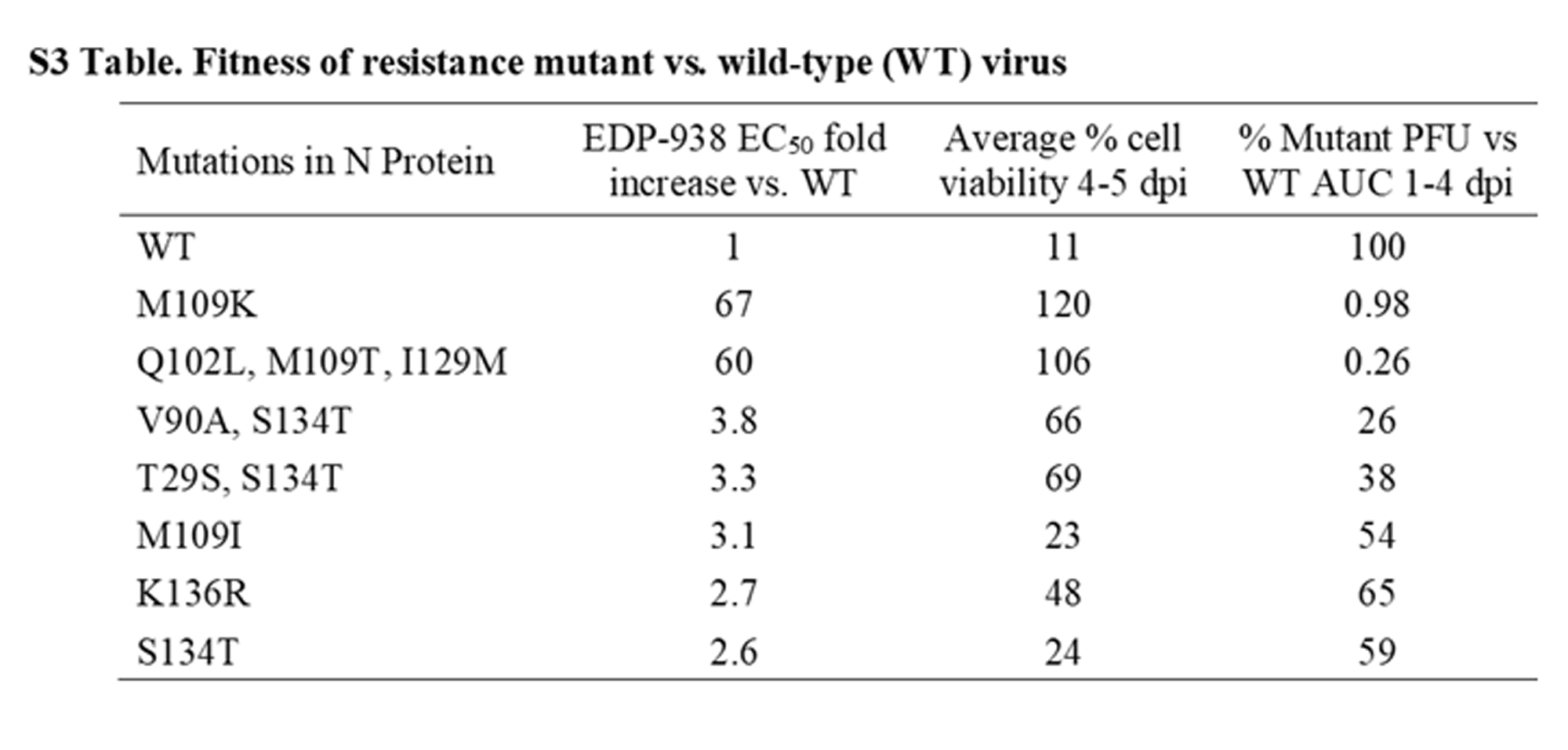

Supplement: S3 Table — (TIF) [file ppat.1009428.s003.tif]

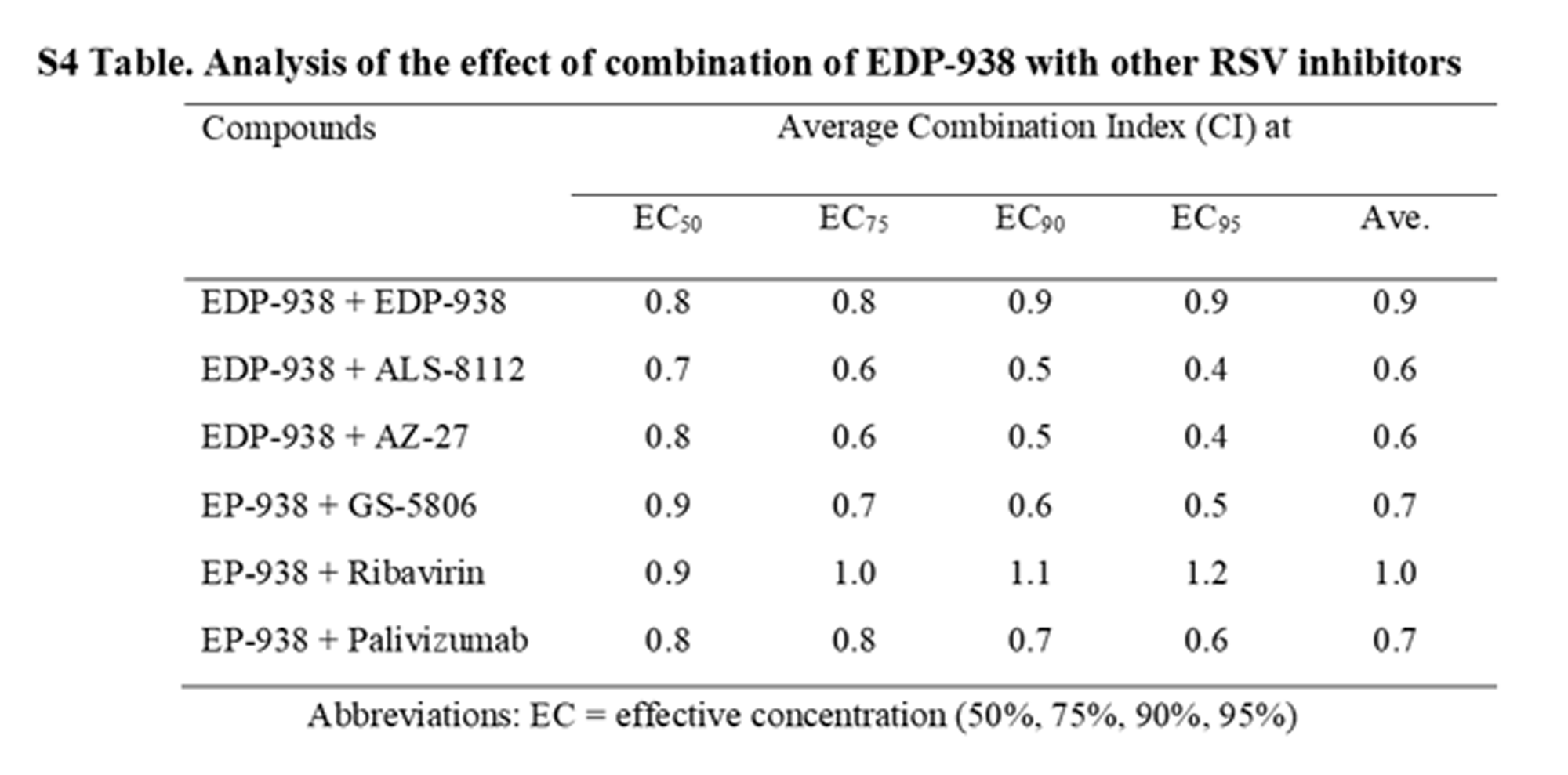

Supplement: S4 Table — (TIF) [file ppat.1009428.s004.tif]

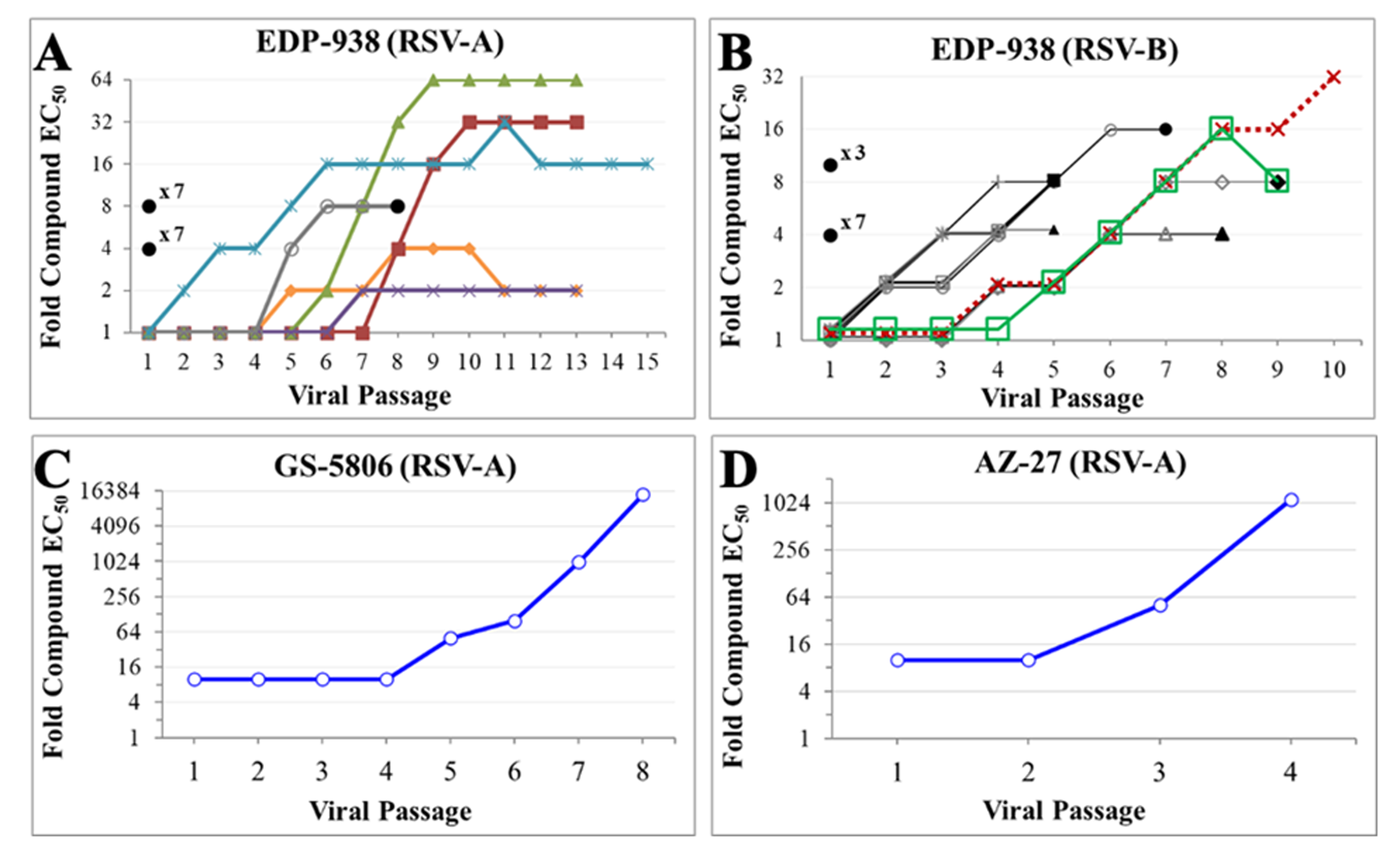

Supplement: S1 Fig — Viral passaging in the presence of increasing concentrations of compound. In each Panel, grayscale curves represent stocks unable to survive with black markers denoting the terminal passage. Resistant strains are indicated by colored lines. (A) RSV-A Long passaged in the presence of EDP-938. 7 attempts to grow virus at 4x and 8x the EDP-938 EC50 value resulted in immediate loss of virus. EDP-938 increases were attempted every passage; all failures but terminal omitted from graph. (B) RSV-B VR-955 passaged. 20 separate attempts resulted in total loss of virus. Some curves slightly offset to display better. (C) GS-5806- and (D) AZ-27-induced viral resistance. Compound increases were not attempted every passage. (TIF) [file ppat.1009428.s005.tif]
